# Outcome and clinical course of EHEC O104 infection in hospitalized patients: A prospective single center study

**DOI:** 10.1371/journal.pone.0191544

**Published:** 2018-02-08

**Authors:** J. P. Albersmeier, J. P. Bremer, W. Dammermann, S. Lüth, F. Hagenmüller, C. Rüther, H. Otto, A. M. Nielsen, U. Schumacher, S. Ullrich

**Affiliations:** 1 Anatomie und Experimentelle Morphologie, Universitätsklinikum Hamburg Eppendorf, Hamburg, Germany; 2 Abteilung für Rheumatologie und Immunology, Klinikum Bad Bramstedt, Bad Bramstedt, Germany; 3 Center of Internal Medicine II, Brandenburg Medical School, Campus Brandenburg a.d.H., Brandenburg an der Havel, Germany; 4 I. Medizinische Klinik, Asklepios Klinik Altona, Hamburg, Germany; Istituto Di Ricerche Farmacologiche Mario Negri, ITALY

## Abstract

**Objectives:**

Shiga-toxin producing O157:H7 Entero Haemorrhagic E. coli [STEC/EHEC] are the most common cause of Haemolytic Uraemic Syndrome [HUS] related to infectious haemorrhagic colitis. Nearly all recommendations on long term treatment of EHEC infections refer to this strain. The 2011 outbreak in Northern Europe was the first of this dimension to be caused by the serotype O104:H4. We report on the 3.5 year follow up of 61 patients diagnosed with symptomatic EHEC O104:H4 infection in spring 2011.

**Methods:**

Patients with EHEC O104 infection were followed in a monocentric, prospective observational study at four time points: 4, 12, 24 and 36 months. These data include the patients’ histories, clinical findings, and complications.

**Results:**

Sixty-one patients suffering from EHEC O104:H4 associated enterocolitis participated in the study at the time of hospital discharge. The mean age of patients was 43 ± 2 years, 37 females and 24 males. 48 patients participated in follow up 1 [FU 1], 34 patients in follow up 2 [FU 2], 23 patients in follow up 3 [FU 3] and 18 patients in follow up 4 [FU 4]. Out of 61 patients discharged from the hospital and included in the study, 54 [84%] were examined at least at one additional follow up. Serum creatinine decreased significantly between discharge and FU 1 from 1.3 ± 0.1 mg/dl to 0.7 ± 0.1 mg/dl [p = 0.0045]. From FU 1 until FU 4, no further change in creatinine levels could be observed. The patients need of antihypertensive medications decreased significantly [p = 0.0005] between discharge and FU 1 after four months. From FU 1 until FU 3, 24 months later, no further significant change in antihypertensive treatment was observed.

**Conclusions:**

Our findings suggest that patients free of pathological findings at time of discharge do not need a specific follow up. Patients with persistent health problems at hospital discharge should be clinically monitored over four months to evaluate chronic organ damage. Progressive or new emerging renal damage could not be observed over time in any patient.

## Introduction

In 2011, Germany experienced the largest recorded outbreak of Entero Hemolytic Escherichia coli [EHEC] O104 associated enterocolitis to date, with fenugreek sprouts from Egypt as a possible causative source of infection [1]. More than 2900 patients were recorded by the Robert Koch Institut [RKI] facing health institutions with a huge battle against this epidemic [1]. The outbreak differed from formerly described outbreaks. It was caused by the O104:H4 strain of E. coli, which was characterized by expression of Shiga-toxin 2 and ‘‘Extended Spectrum b-Lactamase‘‘ [ESBL] [2]. Only two minor outbreaks of EHEC O104:H4 had been reported previously. The largest number of EHEC related outbreaks described so far were associated to the O157:H7 strain [3, 4]. To better outline the genetic specialities of the O104:H4 strain, Brzuszkiewicz et al. proposed the new term Entero Aggregative Hemorrhagic Escherichia coli [EAHEC] [5, 6]. The combination of shiga toxin production, ESBL status and increased adhesive capabilities resulted in unique epidemiologic and clinical findings. The predominance of young, female patients with high complication rates were markedly different to previous episodes of EHEC infections [7].

One of the major complications of EHEC gastroenteritis, Hemolytic Uremic Syndrome [HUS], was experienced by 25% of the patients during this epidemic [8]. HUS, originally described in 1955, is characterized by “the triad of acute renal failure, hemolytic anemia, and thrombocytopenia”[9], typically emerging in children [10]. This outbreak differed from previously described O157:H7 outbreaks concerning the incidence of HUS complication, where it appeared in 7% of mature patients [11]. Female adults were affected the most [68%], while only sporadic cases of infected children were found [8]. Neurological complications occurred with a high prevalence, ranging from seizures, paraplegia to also psychiatric syndromes such as anxiety and hallucinations [12, 13]. Treatment of EHEC related enterocolitis remained symptomatic, while intensified treatment of HUS and HUS related complications by plasmapheresis and dialysis were founded on empiric data, drawn from case series and retrospective analysis. The 2011 EHEC O104:H4 outbreak offered the unique chance to verify these assumptions. Prospective follow up of the acute courses of different patient cohorts resulted in a fundamental change of treatment algorithms. Substitution of intravenous fluids and plasmapheresis represented the therapeutical hallmarks of EHEC related HUS. After short term evaluation of treatment related outcomes of acute HUS, plasmapheresis demonstrated no significant benefit [14]. The previously described negative effect of antibiotic treatment was not observed [15] but instead, selected antibiotic treatment seemed to be beneficial for patients recovery [14].

The vast majority of publications on EHEC related diseases focus on clinical manifestations and courses of the acute phase of the infection [2,6,7]. Only few publications provide detailed follow-up data on well characterized, adult patients and in particular suffering from the O104:H4 strain [16,17]. However, this data is of high interest, as the influence of current treatment concepts have never been evaluated prospectively on long term outcome.

We describe the clinical course of 61 patients with EHEC related hemorrhagic enterocolitis in 2011 over a period of 3.5 years. The manuscript focuses on the long-term outcomes and complications.

## Materials and methods

### Patients

All patients included in this study suffered from EHEC O104:H4 associated enterocolitis during the 2011 outbreak and were treated in a single center as described earlier [7]. First patients were admitted to the hospital on the 14th of May 2011. A total of 61 patients with bloody and /or painful diarrhea due to EHEC O104:H4 enterocolitis were hospitalized. All 61 patients were included in this study. Inclusion criteria were diarrhea (≥3stools/24 h) at time of admission, positive stool testing for EHEC O104:H4 and/ or signs of HUS. HUS was defined as rise of serum creatinine above > 0,5mg/dl, thrombocytopenia <150/nl, signs of hemolysis with anemia, and appearance of schistocytes (Michael M et al, 2009 am Journal Kidney Dis). Patients’ history, treatment and medication, general and abdominal symptoms, physical findings, frequency and quality of stool, blood chemistry, ultrasound, and radiologic findings were collected on admission, discharge, and at the defined time points of follow up. Laboratory data for all patients were recorded at least every second day, in case of HUS daily. All patients gave their written informed consent for this prospective study. The study protocol was approved by the ethical committee of the Chamber of Physicians, Hamburg, Germany [No.: 1123–2011].

### Follow-up and data collection

**Fig 1:**
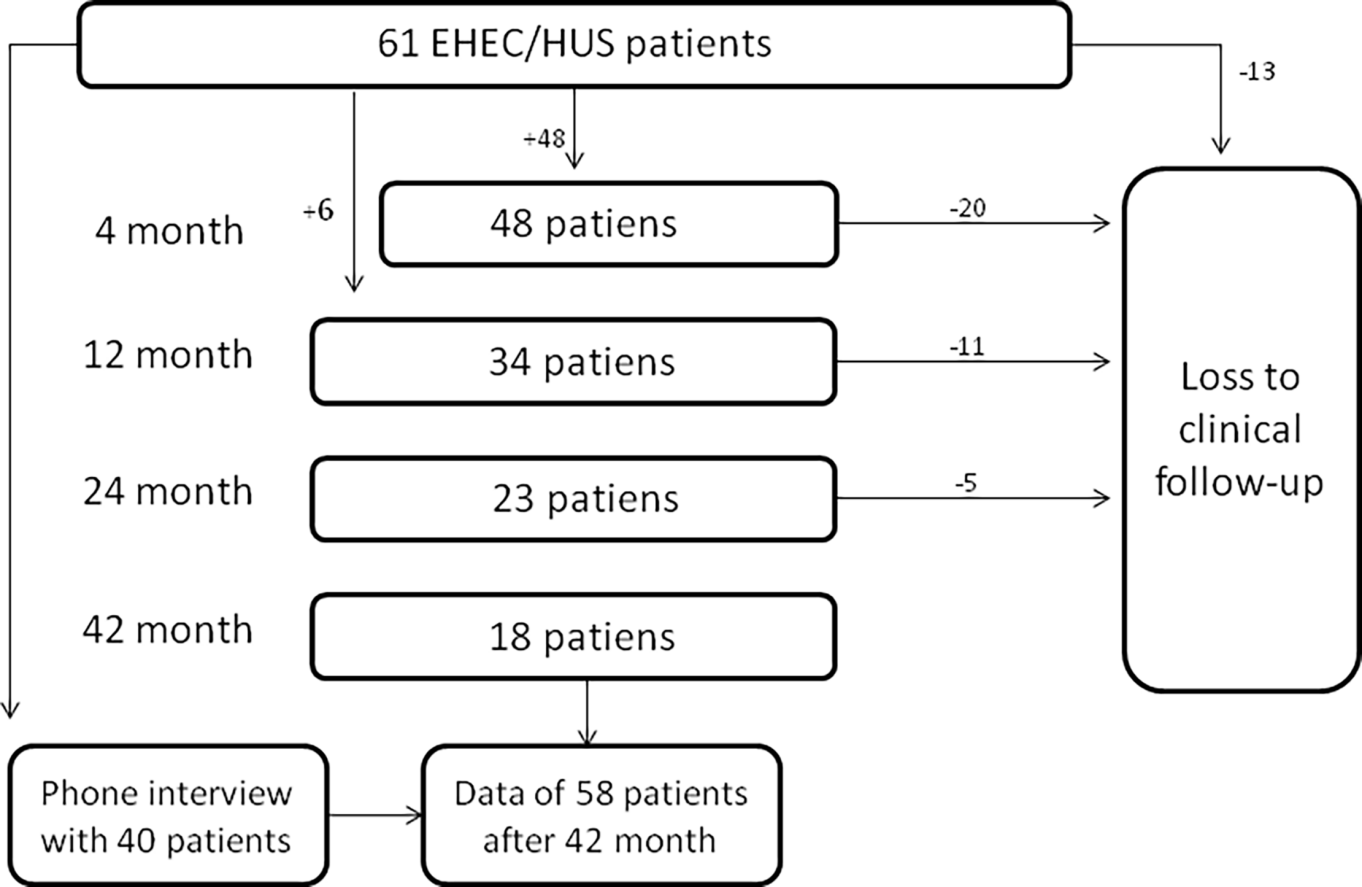
Schematic overview of the follow-up.

Patients were repeatedly seen at four follow-ups [FU] [4, 12, 24 and 42 months after hospital discharge]. Blood, urine and stool samples were collected from each patient and analyzed by the Institut für Klinische Chemie of the Universitätsklinikum Hamburg-Eppendorf and MEDILYS, Laborgesellschaft mbh. Data on the patients’ medical history, drug intake, side effects, physical inspection and blood pressure were documented through a standardized written survey and an additional interview. During all four follow-ups, stool cultures for EHEC were collected three times per patient. Samples were screened for their ESBL presence as well as the presence of Shiga-toxin 1, Shiga-toxin 2, and Intimin-gene using PCR. Further, all stool samples were tested for additional enteropathogenic bacteria and viruses, being other pathogenic E. coli, Salmonella, Campylobacter jejuni, Clostridium, Shigella, and Noro2/Adeno-virus. Patients who did not consistently attend the follow-ups, were interviewed via phone by a standardized questionnaire after 42 months "Fig 1" "S4 Fig".

### Statistical analysis

Clinical data of the study population are described by number of patients and percentage [categorical data] or by mean and SD [continuous data]. Student’s *t*-test was performed to compare datasets. Differences were considered significant at *p<*0.05.

## Results

### Patients

Sixty-one patients were included in the study at the time of hospital discharge. All patients suffered from proven EHEC O104:H4 associated enterocolitis, 59% [n = 36] developed EHEC related HUS during their hospital stay. Patients had no signs for persisting enterocolitis or HUS when discharged from the hospital. Mean age of patients was 43 ± 2 years, 37 females and 24 males. Forty eight patients participated in FU 1 [75%], 34 in FU 2 [56%], 23 in FU 3 [38] and 18 patients in FU 4 [30%]. 54 out of 61 study patients [88%] were examined at least at one time point after discharge. 44 out of 61 study patients [72%] attended at least two follow-ups. The mean number of follow-ups attended by each patient was 2.3. Patients, who did not attend to one of the follow- ups as well as patients who did not attend FU 4, were interviewed by phone after 42 months. Six patients dropped out due to lack of interest for the follow up over time and one patient was not able to communicate properly via phone because of severe neurological damages.

### Patients characteristics at hospital discharge

**Fig 2:**
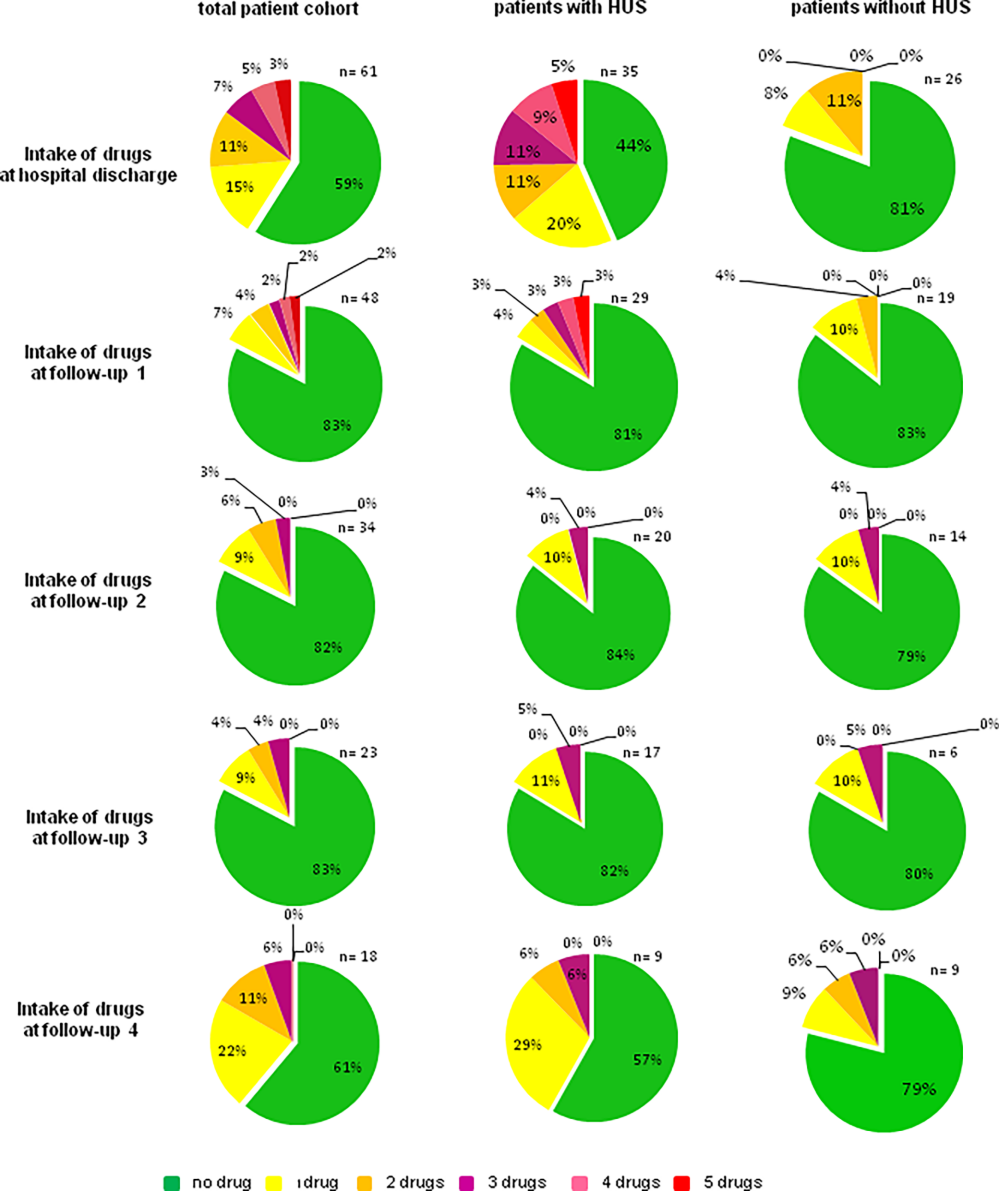
Overview of intake of antihypertensive drugs of patients with EHEC infection. Number of antihypertensive drugs of patients that suffered from EHEC infection with or without HUS, at the time of their hospital discharge.

**Fig 3:**
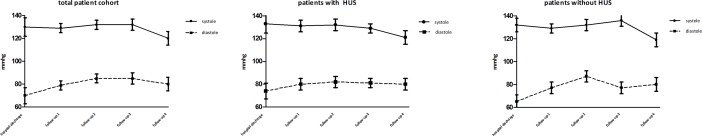
Trend of systolic and diastolic blood pressure. Patients that suffered from EHEC infection with or without HUS, at the time of their hospital discharge. FU 1 (4 month, n = 48), FU 2 (12 month, n = 34), FU 3 (24 month, n = 23) and FU 4 (42 month, n = 18) (mean ± SD).

**Fig 4:**
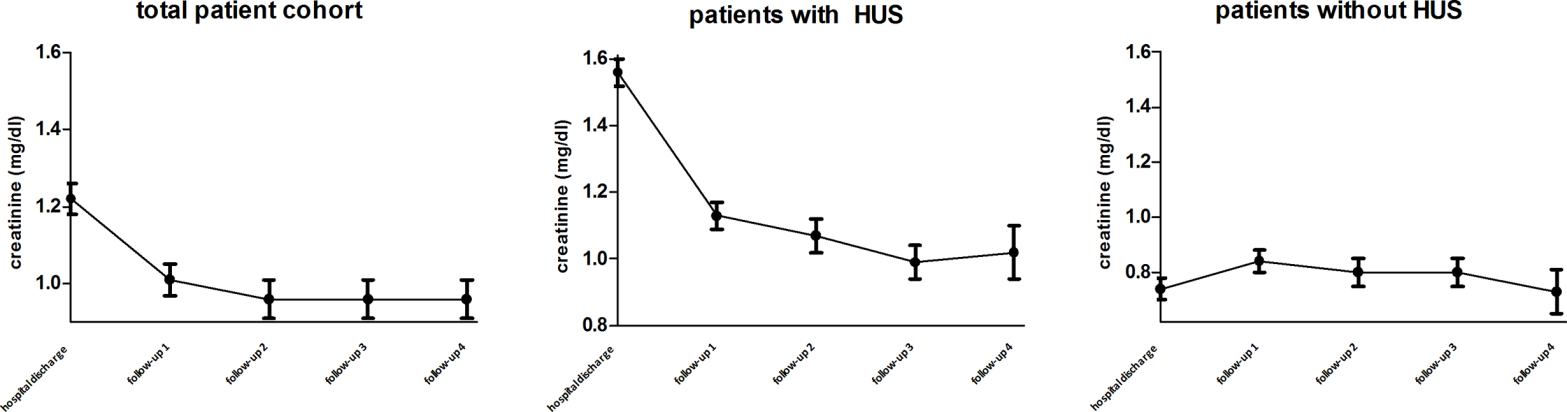
Trend of serum creatinine. Patients that suffered from EHEC infection with or without HUS, at the time of their hospital discharge. FU 1 (4 month, n = 48), FU 2 (12 month, n = 34), FU 3 (24 month, n = 23) and FU 4 (42 month, n = 18) (mean ± SD).

**Fig 5:**
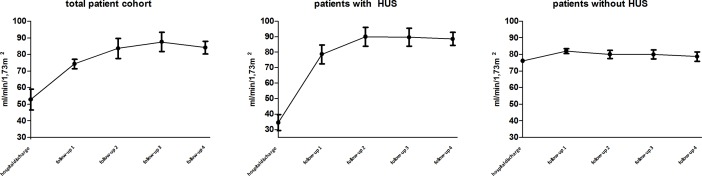
Trend of serum eGFR. Patients that suffered from EHEC infection with or without HUS, at the time of their hospital discharge. FU 1 (4 month, n = 48), FU 2 (12 month, n = 34), FU 3 (24 month, n = 23) and FU 4 (42 month, n = 18) (mean ± SD).

At hospital discharge, 40.1% of the patients [n = 25] took one or more antihypertensive drugs "Fig 2" compared to 13.1% at hospital admission [n = 8] [80% of them suffered from HUS]. The mean systolic blood pressure was 130 ± 3 mmHg, the mean diastolic blood pressure was 77 ± 1 mmHg in all patients at hospital discharge "Fig 3". Mean serum creatinine was 1.3 mg/dl in all patients "Fig 4". Mean eGFR was 52 ml/min/1,73m^2^ in all patients "Fig 5". Patients that suffered from HUS during their hospitalization had higher serum creatinine levels [1,56 mg/dl vs. 0,73 mg/dl], lower eGFR [32 ml/min/1,73m^2^ vs. 75 ml/min/1,73m^2^] and higher need of antihypertensive treatment, compared to patients who did not suffer from HUS [56% vs. 19%] "Figs 2, 4 and 5". One patient suffered from cortical blindness and another patient from severe neurological impairment. In addition most patients [n = 51] had different minor symptoms ranging from abdominal pain to vision disorders. This data has been published earlier [7].

### Prospective follow-up

Forty eight out of 61 patients [75%] were evaluated at FU 1 as described above. The mean age of patients was 40 ± 2, 26 females and 20 males. In all patients serum creatinine had declined significantly after 4 month from 1.3 ± 0.1 mg/dl to 0.7 ± 0,1 mg/dl [p = 0.0045] and eGFR increased from 52 ± 5 ml/min/1,73m^2^ to 74 ± 4 ml/min/1,73m^2^ [p = 0.0012], compared to hospital discharge. In the patient group that suffered from HUS, the decrease of creatinine and increase of eGFR was significantly higher as demonstrated in Figs 4 and 5. Patients that did not suffer from HUS had lower overall creatinine and higher eGFR levels at hospital discharge and four month later no significant reduction could be observed. From follow-up 1 until the last follow-up after 42 month, no further significant changes in creatinine and eGFR levels were observed.

**Fig 6:**
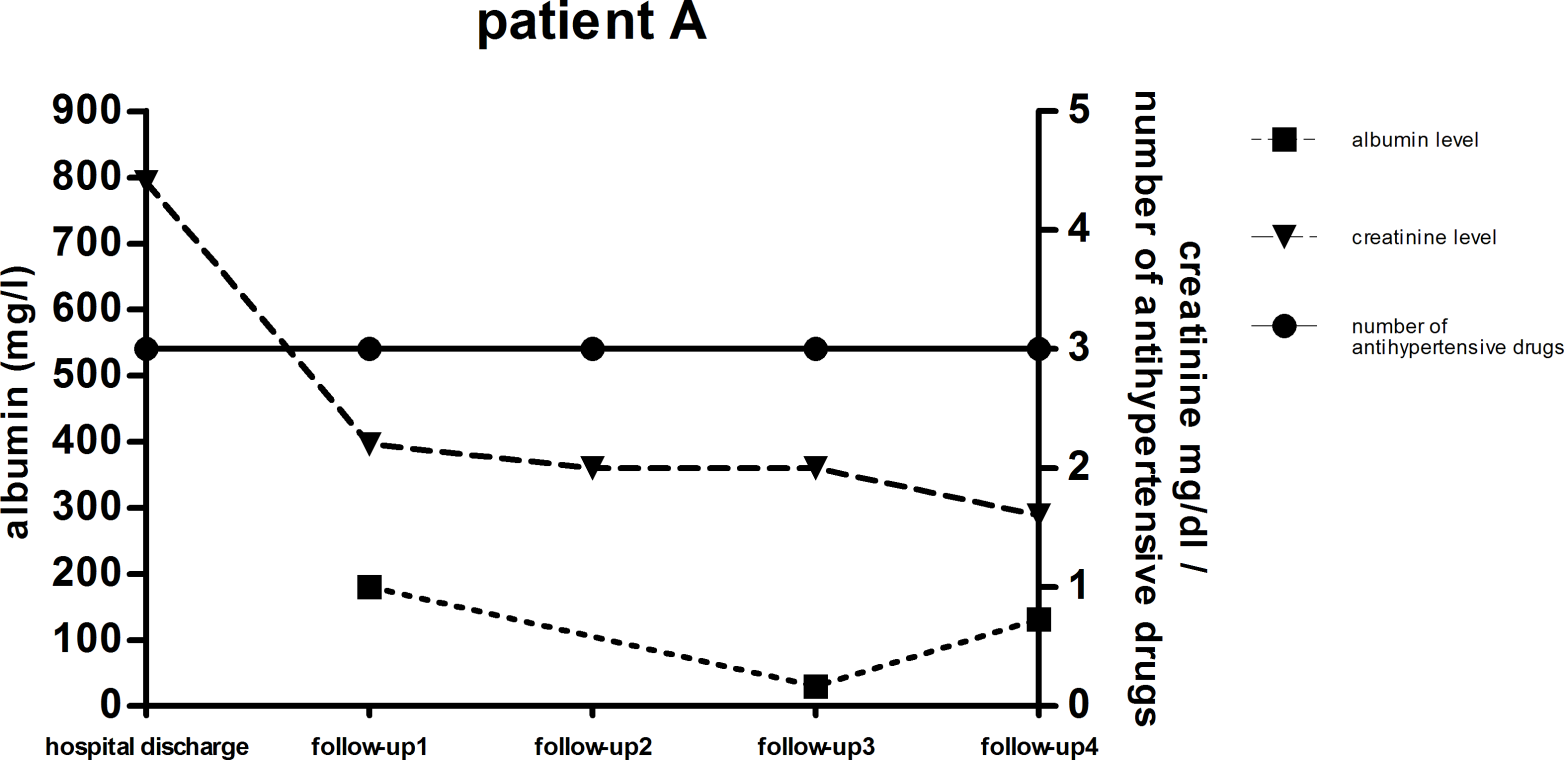
Trend of serum creatinine, urine albumin and antihypertensive drugs. Patient case of severe renal impairment at the time of hospital discharge. FU 1 (4 month, n = 48), FU 2 (12 month, n = 34), FU 3 (24 month, n = 23) and FU 4 (42 month, n = 18).

**Fig 7:**
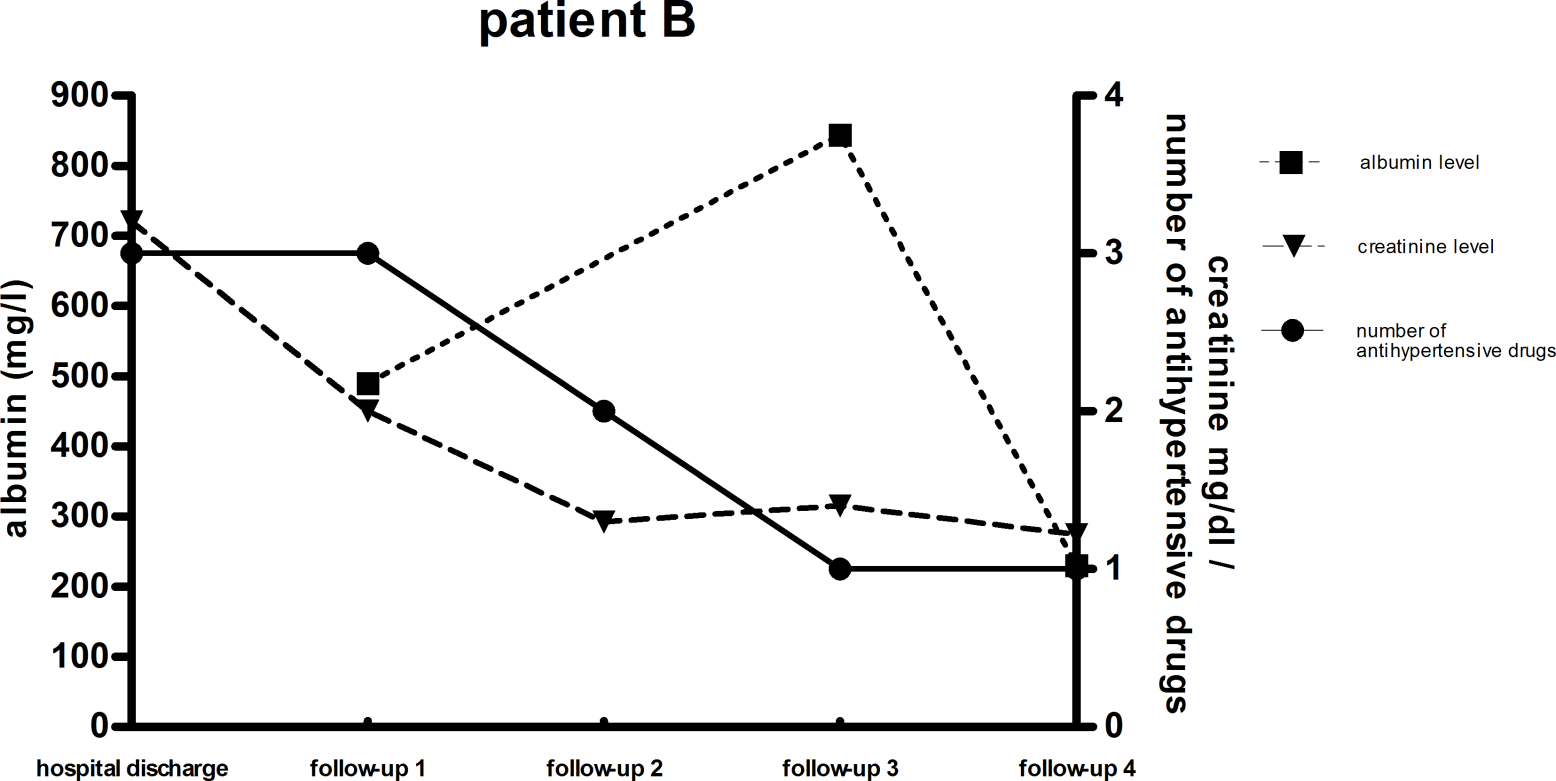
Trend of serum creatinine, urine albumin and antihypertensive drugs. Patient case of severe renal impairment at the time of hospital discharge. FU 1 (4 month, n = 48), FU 2 (12 month, n = 34), FU 3 (24 month, n = 23) and FU 4 (42 month, n = 18).

**Fig 8:**
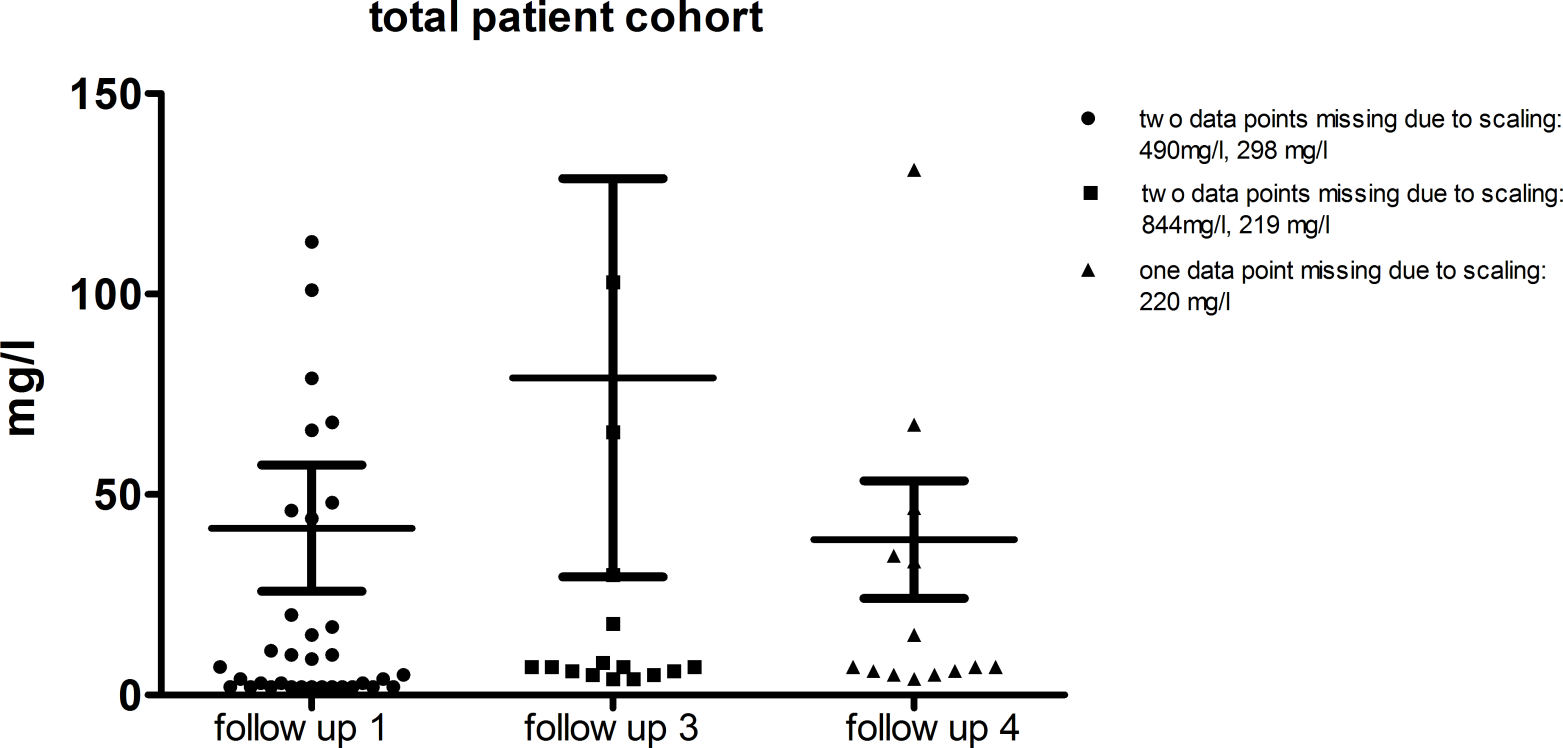
Trend of urine albumin levels of EHEC patients at the time point of their hospital discharge, FU 1 (4 month, n = 48), FU 3 (24 month, n = 23) and FU 4 (42 month, n = 18) (mean ± SD).

The patient’s need of antihypertensive medications dropped significantly [p = 0.0005] over the time period of four months after their release from hospital care. Only 17% [n = 8] of the total patient cohort still needed antihypertensive treatment at follow-up 1 compared to 40.1% [n = 24] at hospital discharge. The need of antihypertensive drug treatment decreased even more in the patients that suffered from HUS infection, from 56% to 19% of the patients in need of antihypertensive drugs at hospital discharge. Patients who did not suffer from HUS, had lower overall need of antihypertensive treatment at both time points [19% vs. 17%] shown in Fig 2. Between FU 1 and 3 no further significant change in antihypertensive treatment was observed [Fig 2]. At FU 4, the need of antihypertensive treatment increased again to 39% of all patients with 43% in the HUS patient group and 22% in the non-HUS patient group. Blood pressure monitoring showed no significant change in systolic and diastolic blood pressure over the whole period from hospital discharge to FU 4, indicating adequate antihypertensive treatment. At hospital discharge, mean blood pressure was 130 ± 3/79 ± 2 mmHg "Fig 3". Four months after hospital discharge, the mean systolic blood pressure was 129 ± 3/79 ± 2 mmHg [p = 0.51; 0,74]. Patients A and B were selected because of the correlation between high urine albumin, high serum creatinine and high blood pressure levels. "Figs 6 and 7" All other patients of the cohort had either normal urine albumin levels, or intermittent albuminuria with normal values on the next FU. The scatter plot of the albuminuria shows the distribution of elevated urine albumin levels, with most patients showing no elevated urine protein levels "Fig 8" "S1–S3 Figs".

Other therapeutic approaches of acute HUS, like plasma-separation, treatment with anti complement factor C5 antibody Eculizumab, and gut restricted antibiotic treatment with Rifaximin during their hospitalization, showed no differences in long term outcome. Many patients suffered from unspecific symptoms like concentration problems and fatigue with a decline of severity over time in both cases [concentration problems: 24% at FU1 vs. 6% at FU4; fatigue 9% at FU1 vs. 6% at FU4]. These findings were subjective data from the patient’s interview and were not quantified. Neurological symptoms did not manifest or aggravate during FU. Severe neurologic deficits after HUS with central nervous system involvement in one patient remained unchanged over time.

## Discussion

This observational study is the first to present data on the long term outcome of patients from the 2011 EHEC O104:H4 outbreak in northern Germany. Patients were observed up to 3.5 years after hospital discharge. A fairly positive outcome on renal function after one year FU has recently been published [18]. The need for longer FU periods was underlined [18, 19]. Our study observed patients from 4 months after hospital discharge up to 3.5 years. Kidney function and the need of antihypertensive treatment were nearly restored to normal after 4 months in both, the HUS and non-HUS patient group. Subsequent follow-up visits up to 3.5 years yielded no further significant changes in clinical and laboratory findings. This finding has to be interpreted with great caution, while the statistics were performed with a simple t test in a simple cohort. Besides this limitation, we still think, the t-test to be the most valuable statistical approach as an ANOVA analysis was not applicable because of the divergent data.

Recommendations for follow up examinations are missing and studies are highly variable in their methods of disease assessment and estimates of long-term prognosis. In a large pediatric cohort, hypertension and renal dysfunction aggravated on the course of 5 years after initial improvement [19]. Therefore our follow-up period could be still too short to draw conclusions on disease related organ damage. A Meta-analysis of a large patient cohort with diarrhea associated HUS without EHEC infection demonstrated that 25% of the patients developed chronic renal damage [20]. A previous study implied that the development of future hypertension correlates with the severity of the HUS syndrome [21]. Accordingly, patients of our cohort suffering from severe EHEC O104:H4 -infections and HUS, also developed aggravated hypertension and reduced kidney function.

In our cohort, renal function of severe and non-severe EAHEC cases had already assimilated at FU 1. Over the following period of 3.5 years, hypertension and renal function remained stable, or improved in all patients. These results are contrary to previous studies [22,23]. Patients suffering from HUS had a significant drop of creatinine levels and a significant increase in eGFR levels after 4 months. Even though creatinine levels remained slightly elevated, no further changes were noted at subsequent follow ups.

Aggravation of neurological disorders did not occur over the course of time in our patient cohort. Patients suffered from a lack of concentration and general fatigue. These conditions improved over time. These findings match with recently published data, concluding that long term sequelae of EHEC O104:H4 associated neurological impairment in adults is rather positive [24].

One limitation of our long time follow-up was the inconsistent attendance to clinical visits: Out of 61 patients discharged from hospital and included in the study, 54 [88%] were examined at least once. Only 18 patients [29,5%] attended the last FU after 3,5 years. The mean number of follow-ups attended by each patient was 2.3. Patients who were free of symptoms were more often lost to follow up, compared to those with persisting complaints. However, regarding fatal outcome or severe complications, no differences were found between these two groups. Another limitation of our study was the rather small sample size, which excluded multivariate analyses. However we observed a clinical bias by patients with severe courses of disease being more compliant to follow-ups. Another limitation is the observational approach of the study. As no data was collected in a prospective setting, clinical data cannot be compared directly.

We conclude that during acute EHEC O104:H4 -infection, clinicians should focus on the multidisciplinary patient care, as described earlier [7]. Patients with normalized renal function and a good general state of health, do not need necessarily any further follow-up. Patients, who are discharged with manifest organ damage, need a close monitoring during the first four month to estimate the further health outcome. Patients with severe complications during EHEC O104:H4 -infection should be monitored longer with an individual time span to evaluate long term complications.

## Supporting information

S1 Fig Statistical analysis. (DOCX)

S2 Fig Patient data at hospital discharge. (XLS)

S3 Fig Patient data at follow-ups. (XLS)

S4 Fig Patients interviews by telephone. (TIF)
