# Supplementary material for: Outcome and clinical course of EHEC O104 infection in hospitalized patients: A prospective single center study
Source: PLoS One. 2018 Feb 8;13(2):e0191544. doi: 10.1371/journal.pone.0191544 (PMC5805174; doi:10.1371/journal.pone.0191544)
Supplement: S1 Fig — (DOCX) [file pone.0191544.s001.docx]

| **Statistical analysis: .......................................................** |
| --- |
|  |
| **Analyse Kreatinin** |
| **Analyse 1: Analysis all Patients** |

| ***The MEANS Procedure*** |
| --- |

| **Variable** | **N** | **N Miss** | **Minimum** | **Lower Quartile** | **Median** | **Upper Quartile** | **Maximum** |
| --- | --- | --- | --- | --- | --- | --- | --- |
| KREA1 KREA2 KREA3 KREA4 KREA5 KREA6 | 61 60 46 33 23 18 | 0 1 15 28 38 43 | 0.60 0.50 0.70 0.60 0.50 0.60 | 0.70 0.70 0.80 0.80 0.80 0.70 | 0.80 0.80 0.90 0.90 0.90 0.90 | 1.10 1.20 1.00 1.00 1.10 1.00 | 5.80 4.80 3.60 1.90 2.00 1.60 |

| **Analyse 1: Analysis Patients at FU 4** |
| --- |

***The SAS System***

| ***The MEANS Procedure*** |
| --- |

| **Variable** | **N** | **N Miss** | **Minimum** | **Lower Quartile** | **Median** | **Upper Quartile** | **Maximum** |
| --- | --- | --- | --- | --- | --- | --- | --- |
| KREA1 KREA2 KREA3 KREA4 KREA5 KREA6 | 18 18 18 17 18 18 | 0 0 0 1 0 0 | 0.60 0.50 0.70 0.60 0.50 0.60 | 0.70 0.70 0.70 0.70 0.80 0.70 | 0.80 1.10 0.80 0.90 0.80 0.90 | 1.10 2.00 1.10 1.10 1.00 1.00 | 3.90 4.40 2.20 1.90 2.00 1.60 |

| **Analyse 1: Analysis Patients at FU 3** |
| --- |

***The SAS System***

| ***The MEANS Procedure*** |
| --- |

| **Variable** | **N** | **N Miss** | **Minimum** | **Lower Quartile** | **Median** | **Upper Quartile** | **Maximum** |
| --- | --- | --- | --- | --- | --- | --- | --- |
| KREA1 KREA2 KREA3 KREA4 KREA5 KREA6 | 23 23 21 22 23 18 | 0 0 2 1 0 5 | 0.60 0.50 0.70 0.60 0.50 0.60 | 0.70 0.70 0.70 0.80 0.80 0.70 | 0.80 1.20 0.90 0.90 0.90 0.90 | 1.60 2.00 1.20 1.10 1.10 1.00 | 3.90 4.40 2.20 1.90 2.00 1.60 |

| **Analyse 1: Analysis Patients at FU 2** |
| --- |

***The SAS System***

| ***The MEANS Procedure*** |
| --- |

| **Variable** | **N** | **N Miss** | **Minimum** | **Lower Quartile** | **Median** | **Upper Quartile** | **Maximum** |
| --- | --- | --- | --- | --- | --- | --- | --- |
| KREA1 KREA2 KREA3 KREA4 KREA5 KREA6 | 33 33 30 33 22 17 | 0 0 3 0 11 16 | 0.60 0.50 0.70 0.60 0.50 0.60 | 0.70 0.70 0.80 0.80 0.80 0.70 | 0.90 1.00 0.90 0.90 0.90 0.90 | 1.50 1.50 1.10 1.00 1.10 1.00 | 3.90 4.40 2.20 1.90 2.00 1.60 |

| **Analyse 1: Analysis Patients at FU 1** |
| --- |

***The SAS System***

| ***The MEANS Procedure*** |
| --- |

| **Variable** | **N** | **N Miss** | **Minimum** | **Lower Quartile** | **Median** | **Upper Quartile** | **Maximum** |
| --- | --- | --- | --- | --- | --- | --- | --- |
| KREA1 KREA2 KREA3 KREA4 KREA5 KREA6 | 46 46 46 30 21 18 | 0 0 0 16 25 28 | 0.60 0.50 0.70 0.60 0.50 0.60 | 0.70 0.70 0.80 0.80 0.80 0.70 | 0.80 0.80 0.90 0.90 0.90 0.90 | 1.10 1.20 1.00 1.10 1.10 1.00 | 3.90 4.80 3.60 1.90 2.00 1.60 |

| **Analyse 2: HOSPITAL ADMISSION VERSUS DISCHARGE** |
| --- |

***The SAS System***

| ***The MEANS Procedure*** |
| --- |

| **Variable** | **N** | **N Miss** | **Minimum** | **Lower Quartile** | **Median** | **Upper Quartile** | **Maximum** |
| --- | --- | --- | --- | --- | --- | --- | --- |
| KREA1 KREA2 | 60 60 | 0 0 | 0.60 0.50 | 0.70 0.70 | 0.80 0.80 | 1.10 1.20 | 5.80 4.80 |

***The SAS System***

| ***The UNIVARIATE Procedure*** | |
| --- | --- |
| ***Variable: K21*** |  |

| **Tests for Location: Mu0=0** | | | | |
| --- | --- | --- | --- | --- |
| **Test** | **Statistic** | | **p Value** | |
| **Student's t** | **t** | -0.18649 | **Pr > \|t\|** | 0.8527 |
| **Sign** | **M** | -6 | **Pr >= \|M\|** | 0.1114 |
| **Signed Rank** | **S** | -109 | **Pr >= \|S\|** | 0.2674 |

| **Analyse 2: Hospital Discharge VERSUS FU1** |
| --- |

***The SAS System***

| ***The MEANS Procedure*** |
| --- |

| **Variable** | **N** | **N Miss** | **Minimum** | **Lower Quartile** | **Median** | **Upper Quartile** | **Maximum** |
| --- | --- | --- | --- | --- | --- | --- | --- |
| KREA2 KREA3 | 46 46 | 0 0 | 0.50 0.70 | 0.70 0.80 | 0.80 0.90 | 1.20 1.00 | 4.80 3.60 |

***The SAS System***

| ***The UNIVARIATE Procedure*** | |
| --- | --- |
| ***Variable: K32*** |  |

| **Tests for Location: Mu0=0** | | | | |
| --- | --- | --- | --- | --- |
| **Test** | **Statistic** | | **p Value** | |
| **Student's t** | **t** | -2.98748 | **Pr > \|t\|** | 0.0045 |
| **Sign** | **M** | -0.5 | **Pr >= \|M\|** | 1.0000 |
| **Signed Rank** | **S** | -116 | **Pr >= \|S\|** | 0.0551 |

| **Analyse 2: FU1 VERSUS FU2** |
| --- |

***The SAS System***

| ***The MEANS Procedure*** |
| --- |

| **Variable** | **N** | **N Miss** | **Minimum** | **Lower Quartile** | **Median** | **Upper Quartile** | **Maximum** |
| --- | --- | --- | --- | --- | --- | --- | --- |
| KREA3 KREA4 | 30 30 | 0 0 | 0.70 0.60 | 0.80 0.80 | 0.90 0.90 | 1.10 1.10 | 2.20 1.90 |

***The SAS System***

| ***The UNIVARIATE Procedure*** | |
| --- | --- |
| ***Variable: K43*** |  |

| **Tests for Location: Mu0=0** | | | | |
| --- | --- | --- | --- | --- |
| **Test** | **Statistic** | | **p Value** | |
| **Student's t** | **t** | -1.43583 | **Pr > \|t\|** | 0.1618 |
| **Sign** | **M** | -1.5 | **Pr >= \|M\|** | 0.6476 |
| **Signed Rank** | **S** | -32.5 | **Pr >= \|S\|** | 0.1935 |

| **Analyse 2: FU2 VERSUS FU3** |
| --- |

***The SAS System***

| ***The MEANS Procedure*** |
| --- |

| **Variable** | **N** | **N Miss** | **Minimum** | **Lower Quartile** | **Median** | **Upper Quartile** | **Maximum** |
| --- | --- | --- | --- | --- | --- | --- | --- |
| KREA4 KREA5 | 22 22 | 0 0 | 0.60 0.50 | 0.80 0.80 | 0.90 0.90 | 1.10 1.10 | 1.90 2.00 |

***The SAS System***

| ***The UNIVARIATE Procedure*** | |
| --- | --- |
| ***Variable: K54*** |  |

| **Tests for Location: Mu0=0** | | | | |
| --- | --- | --- | --- | --- |
| **Test** | **Statistic** | | **p Value** | |
| **Student's t** | **t** | -0.59106 | **Pr > \|t\|** | 0.5608 |
| **Sign** | **M** | -2 | **Pr >= \|M\|** | 0.4545 |
| **Signed Rank** | **S** | 1 | **Pr >= \|S\|** | 0.9668 |

| **Analyse 2: FU3 VERSUS FU4** |
| --- |

***The SAS System***

| ***The MEANS Procedure*** |
| --- |

| **Variable** | **N** | **N Miss** | **Minimum** | **Lower Quartile** | **Median** | **Upper Quartile** | **Maximum** |
| --- | --- | --- | --- | --- | --- | --- | --- |
| KREA5 KREA6 | 18 18 | 0 0 | 0.50 0.60 | 0.80 0.70 | 0.80 0.90 | 1.00 1.00 | 2.00 1.60 |

***The SAS System***

| ***The UNIVARIATE Procedure*** | |
| --- | --- |
| ***Variable: K65*** |  |

| **Tests for Location: Mu0=0** | | | | |
| --- | --- | --- | --- | --- |
| **Test** | **Statistic** | | **p Value** | |
| **Student's t** | **t** | 0.368782 | **Pr > \|t\|** | 0.7168 |
| **Sign** | **M** | 0.5 | **Pr >= \|M\|** | 1.0000 |
| **Signed Rank** | **S** | -3.5 | **Pr >= \|S\|** | 0.8269 |

| **Analysis Bloodpressure medication** |  |
| --- | --- |
|  |  |
| **Analyse 1: Analysis all Patients** | |

***The SAS System***

| ***The MEANS Procedure*** |
| --- |

| **Variable** | **N** | **N Miss** | **Minimum** | **Lower Quartile** | **Median** | **Upper Quartile** | **Maximum** |
| --- | --- | --- | --- | --- | --- | --- | --- |
| RRMEDIS1 RRMEDIS2 RRMEDIS3 RRMEDIS4 RRMEDIS5 RRMEDIS6 | 61 60 46 29 23 18 | 0 1 15 32 38 43 | 0.00 0.00 0.00 0.00 0.00 0.00 | 0.00 0.00 0.00 0.00 0.00 0.00 | 0.00 0.00 0.00 0.00 0.00 0.00 | 0.00 2.00 0.00 0.00 0.00 1.00 | 4.00 5.00 5.00 3.00 3.00 3.00 |

| **Analyse 1: Analysis patients at FU 4** |
| --- |

***The SAS System***

| ***The MEANS Procedure*** |
| --- |

| **Variable** | **N** | **N Miss** | **Minimum** | **Lower Quartile** | **Median** | **Upper Quartile** | **Maximum** |
| --- | --- | --- | --- | --- | --- | --- | --- |
| RRMEDIS1 RRMEDIS2 RRMEDIS3 RRMEDIS4 RRMEDIS5 RRMEDIS6 | 18 18 18 17 18 18 | 0 0 0 1 0 0 | 0.00 0.00 0.00 0.00 0.00 0.00 | 0.00 0.00 0.00 0.00 0.00 0.00 | 0.00 0.50 0.00 0.00 0.00 0.00 | 0.00 1.00 0.00 0.00 0.00 1.00 | 2.00 4.00 4.00 3.00 3.00 3.00 |

| **Analyse 1: Analysis patients at FU 3** |
| --- |

***The SAS System***

| ***The MEANS Procedure*** |
| --- |

| **Variable** | **N** | **N Miss** | **Minimum** | **Lower Quartile** | **Median** | **Upper Quartile** | **Maximum** |
| --- | --- | --- | --- | --- | --- | --- | --- |
| RRMEDIS1 RRMEDIS2 RRMEDIS3 RRMEDIS4 RRMEDIS5 RRMEDIS6 | 23 23 22 20 23 18 | 0 0 1 3 0 5 | 0.00 0.00 0.00 0.00 0.00 0.00 | 0.00 0.00 0.00 0.00 0.00 0.00 | 0.00 1.00 0.00 0.00 0.00 0.00 | 0.00 2.00 0.00 0.00 0.00 1.00 | 3.00 5.00 5.00 3.00 3.00 3.00 |

| **Analyse 1: Analysis patients at FU 2** |
| --- |

***The SAS System***

| ***The MEANS Procedure*** |
| --- |

| **Variable** | **N** | **N Miss** | **Minimum** | **Lower Quartile** | **Median** | **Upper Quartile** | **Maximum** |
| --- | --- | --- | --- | --- | --- | --- | --- |
| RRMEDIS1 RRMEDIS2 RRMEDIS3 RRMEDIS4 RRMEDIS5 RRMEDIS6 | 29 29 29 29 20 17 | 0 0 0 0 9 12 | 0.00 0.00 0.00 0.00 0.00 0.00 | 0.00 0.00 0.00 0.00 0.00 0.00 | 0.00 0.00 0.00 0.00 0.00 0.00 | 0.00 1.00 0.00 0.00 0.00 1.00 | 2.00 4.00 4.00 3.00 3.00 3.00 |

| **Analyse 1: Analysis patients at FU 1** |
| --- |

***The SAS System***

| ***The MEANS Procedure*** |
| --- |

| **Variable** | **N** | **N Miss** | **Minimum** | **Lower Quartile** | **Median** | **Upper Quartile** | **Maximum** |
| --- | --- | --- | --- | --- | --- | --- | --- |
| RRMEDIS1 RRMEDIS2 RRMEDIS3 RRMEDIS4 RRMEDIS5 RRMEDIS6 | 46 46 46 29 22 18 | 0 0 0 17 24 28 | 0.00 0.00 0.00 0.00 0.00 0.00 | 0.00 0.00 0.00 0.00 0.00 0.00 | 0.00 0.00 0.00 0.00 0.00 0.00 | 0.00 1.00 0.00 0.00 0.00 1.00 | 3.00 5.00 5.00 3.00 3.00 3.00 |

| **Analyse 2: HOSPITAL ADMISSION VERSUS DISCHARGE** |
| --- |

***The SAS System***

| ***The MEANS Procedure*** |
| --- |

| **Variable** | **N** | **N Miss** | **Minimum** | **Lower Quartile** | **Median** | **Upper Quartile** | **Maximum** |
| --- | --- | --- | --- | --- | --- | --- | --- |
| RRMEDIS1 RRMEDIS2 | 60 60 | 0 0 | 0.00 0.00 | 0.00 0.00 | 0.00 0.00 | 0.00 2.00 | 4.00 5.00 |

***The SAS System***

| ***The UNIVARIATE Procedure*** | |
| --- | --- |
| ***Variable: RM21*** |  |

| **Tests for Location: Mu0=0** | | | | |
| --- | --- | --- | --- | --- |
| **Test** | **Statistic** | | **p Value** | |
| **Student's t** | **t** | 4.475491 | **Pr > \|t\|** | <.0001 |
| **Sign** | **M** | 10 | **Pr >= \|M\|** | <.0001 |
| **Signed Rank** | **S** | 105 | **Pr >= \|S\|** | <.0001 |

| **Analyse 2: DISCHARGE VERSUS FU1** |
| --- |

***The SAS System***

| ***The MEANS Procedure*** |
| --- |

| **Variable** | **N** | **N Miss** | **Minimum** | **Lower Quartile** | **Median** | **Upper Quartile** | **Maximum** |
| --- | --- | --- | --- | --- | --- | --- | --- |
| RRMEDIS2 RRMEDIS3 | 46 46 | 0 0 | 0.00 0.00 | 0.00 0.00 | 0.00 0.00 | 1.00 0.00 | 5.00 5.00 |

***The SAS System***

| ***The UNIVARIATE Procedure*** | |
| --- | --- |
| ***Variable: RM32*** |  |

| **Tests for Location: Mu0=0** | | | | |
| --- | --- | --- | --- | --- |
| **Test** | **Statistic** | | **p Value** | |
| **Student's t** | **t** | -2.93892 | **Pr > \|t\|** | 0.0052 |
| **Sign** | **M** | -5.5 | **Pr >= \|M\|** | 0.0074 |
| **Signed Rank** | **S** | -46 | **Pr >= \|S\|** | 0.0065 |

| **Analyse 2: FU1 VERSUS FU2** |
| --- |

***The SAS System***

| ***The MEANS Procedure*** |
| --- |

| **Variable** | **N** | **N Miss** | **Minimum** | **Lower Quartile** | **Median** | **Upper Quartile** | **Maximum** |
| --- | --- | --- | --- | --- | --- | --- | --- |
| RRMEDIS3 RRMEDIS4 | 29 29 | 0 0 | 0.00 0.00 | 0.00 0.00 | 0.00 0.00 | 0.00 0.00 | 4.00 3.00 |

***The SAS System***

| ***The UNIVARIATE Procedure*** | |
| --- | --- |
| ***Variable: RM43*** |  |

| **Tests for Location: Mu0=0** | | | | |
| --- | --- | --- | --- | --- |
| **Test** | **Statistic** | | **p Value** | |
| **Student's t** | **t** | -0.32817 | **Pr > \|t\|** | 0.7452 |
| **Sign** | **M** | 0 | **Pr >= \|M\|** | 1.0000 |
| **Signed Rank** | **S** | -1.5 | **Pr >= \|S\|** | 1.0000 |

| **Analyse 2: FU2 VERSUS FU3** |
| --- |

***The SAS System***

| ***The MEANS Procedure*** |
| --- |

| **Variable** | **N** | **N Miss** | **Minimum** | **Lower Quartile** | **Median** | **Upper Quartile** | **Maximum** |
| --- | --- | --- | --- | --- | --- | --- | --- |
| RRMEDIS4 RRMEDIS5 | 20 20 | 0 0 | 0.00 0.00 | 0.00 0.00 | 0.00 0.00 | 0.00 0.00 | 3.00 3.00 |

***The SAS System***

| ***The UNIVARIATE Procedure*** | |
| --- | --- |
| ***Variable: RM54*** |  |

| **Tests for Location: Mu0=0** | | | | |
| --- | --- | --- | --- | --- |
| **Test** | **Statistic** | | **p Value** | |
| **Student's t** | **t** | 0.56748 | **Pr > \|t\|** | 0.5770 |
| **Sign** | **M** | 0.5 | **Pr >= \|M\|** | 1.0000 |
| **Signed Rank** | **S** | 1 | **Pr >= \|S\|** | 1.0000 |

| **Analyse 2: FU3 VERSUS FU4** |
| --- |

***The SAS System***

| ***The MEANS Procedure*** |
| --- |

| **Variable** | **N** | **N Miss** | **Minimum** | **Lower Quartile** | **Median** | **Upper Quartile** | **Maximum** |
| --- | --- | --- | --- | --- | --- | --- | --- |
| RRMEDIS5 RRMEDIS6 | 18 18 | 0 0 | 0.00 0.00 | 0.00 0.00 | 0.00 0.00 | 0.00 1.00 | 3.00 3.00 |

***The SAS System***

| ***The UNIVARIATE Procedure*** | |
| --- | --- |
| ***Variable: RM65*** |  |

| **Tests for Location: Mu0=0** | | | | |
| --- | --- | --- | --- | --- |
| **Test** | **Statistic** | | **p Value** | |
| **Student's t** | **t** | 2.203893 | **Pr > \|t\|** | 0.0416 |
| **Sign** | **M** | 2 | **Pr >= \|M\|** | 0.1250 |
| **Signed Rank** | **S** | 5 | **Pr >= \|S\|** | 0.1250 |

| **Analysis systolic bloodpressure** |  |
| --- | --- |
|  |  |
| **Analyse 1: Analysis all Patients** | |

***The SAS System***

| ***The MEANS Procedure*** |
| --- |

| **Variable** | **N** | **N Miss** | **Minimum** | **Lower Quartile** | **Median** | **Upper Quartile** | **Maximum** |
| --- | --- | --- | --- | --- | --- | --- | --- |
| RRSYST1 RRSYST2 RRSYST3 RRSYST4 RRSYST5 RRSYST6 | 61 52 44 29 15 17 | 0 9 17 32 46 44 | 90.00 90.00 100.00 98.00 110.00 100.00 | 120.00 120.00 120.00 120.00 122.00 113.00 | 130.00 130.00 130.00 134.00 131.00 130.00 | 150.00 140.00 140.00 143.00 139.00 134.00 | 170.00 180.00 180.00 160.00 170.00 160.00 |

| **Analyse 1: Analysis Patients at FU 4** |
| --- |

***The SAS System***

| ***The MEANS Procedure*** |
| --- |

| **Variable** | **N** | **N Miss** | **Minimum** | **Lower Quartile** | **Median** | **Upper Quartile** | **Maximum** |
| --- | --- | --- | --- | --- | --- | --- | --- |
| RRSYST1 RRSYST2 RRSYST3 RRSYST4 RRSYST5 RRSYST6 | 17 14 17 16 14 17 | 0 3 0 1 3 0 | 110.00 110.00 100.00 98.00 110.00 100.00 | 120.00 120.00 120.00 117.00 122.00 113.00 | 130.00 125.00 130.00 132.00 133.00 130.00 | 146.00 150.00 140.00 139.50 139.00 134.00 | 160.00 180.00 165.00 160.00 170.00 160.00 |

| **Analyse 1: Analysis Patients at FU 3** |
| --- |

***The SAS System***

| ***The MEANS Procedure*** |
| --- |

| **Variable** | **N** | **N Miss** | **Minimum** | **Lower Quartile** | **Median** | **Upper Quartile** | **Maximum** |
| --- | --- | --- | --- | --- | --- | --- | --- |
| RRSYST1 RRSYST2 RRSYST3 RRSYST4 RRSYST5 RRSYST6 | 15 12 15 14 15 14 | 0 3 0 1 0 1 | 110.00 110.00 100.00 98.00 110.00 100.00 | 127.00 115.00 115.00 120.00 122.00 107.00 | 130.00 125.00 130.00 132.00 131.00 128.50 | 149.00 140.00 140.00 139.00 139.00 134.00 | 160.00 160.00 160.00 154.00 170.00 160.00 |

| **Analyse 1: Analysis Patients at FU 2** |
| --- |

***The SAS System***

| ***The MEANS Procedure*** |
| --- |

| **Variable** | **N** | **N Miss** | **Minimum** | **Lower Quartile** | **Median** | **Upper Quartile** | **Maximum** |
| --- | --- | --- | --- | --- | --- | --- | --- |
| RRSYST1 RRSYST2 RRSYST3 RRSYST4 RRSYST5 RRSYST6 | 29 24 28 29 14 16 | 0 5 1 0 15 13 | 100.00 110.00 100.00 98.00 110.00 100.00 | 120.00 120.00 120.00 120.00 124.00 110.00 | 136.00 130.00 130.00 134.00 133.00 128.50 | 149.00 150.00 140.00 143.00 139.00 138.00 | 160.00 180.00 165.00 160.00 170.00 160.00 |

| **Analyse 1: Analysis Patients at FU 1** |
| --- |

***The SAS System***

| ***The MEANS Procedure*** |
| --- |

| **Variable** | **N** | **N Miss** | **Minimum** | **Lower Quartile** | **Median** | **Upper Quartile** | **Maximum** |
| --- | --- | --- | --- | --- | --- | --- | --- |
| RRSYST1 RRSYST2 RRSYST3 RRSYST4 RRSYST5 RRSYST6 | 44 39 44 28 15 17 | 0 5 0 16 29 27 | 90.00 90.00 100.00 98.00 110.00 100.00 | 120.00 120.00 120.00 120.00 122.00 113.00 | 130.00 130.00 130.00 134.00 131.00 130.00 | 150.00 140.00 140.00 143.50 139.00 134.00 | 170.00 180.00 180.00 160.00 170.00 160.00 |

| **Analyse 2: HOSPITAL ADMISSION VERSUS DISCHARGE** |
| --- |

***The SAS System***

| ***The MEANS Procedure*** |
| --- |

| **Variable** | **N** | **N Miss** | **Minimum** | **Lower Quartile** | **Median** | **Upper Quartile** | **Maximum** |
| --- | --- | --- | --- | --- | --- | --- | --- |
| RRSYST1 RRSYST2 | 52 52 | 0 0 | 90.00 90.00 | 120.00 120.00 | 131.00 130.00 | 147.50 140.00 | 170.00 180.00 |

***The SAS System***

| ***The UNIVARIATE Procedure*** | |
| --- | --- |
| ***Variable: RS21*** |  |

| **Tests for Location: Mu0=0** | | | | |
| --- | --- | --- | --- | --- |
| **Test** | **Statistic** | | **p Value** | |
| **Student's t** | **t** | -1.32443 | **Pr > \|t\|** | 0.1913 |
| **Sign** | **M** | -3.5 | **Pr >= \|M\|** | 0.3713 |
| **Signed Rank** | **S** | -107.5 | **Pr >= \|S\|** | 0.2278 |

| **Analyse 2: ENTLASSUNG VERSUS FU1** |
| --- |

***The SAS System***

| ***The MEANS Procedure*** |
| --- |

| **Variable** | **N** | **N Miss** | **Minimum** | **Lower Quartile** | **Median** | **Upper Quartile** | **Maximum** |
| --- | --- | --- | --- | --- | --- | --- | --- |
| RRSYST2 RRSYST3 | 39 39 | 0 0 | 90.00 100.00 | 120.00 120.00 | 130.00 130.00 | 140.00 140.00 | 180.00 180.00 |

***The SAS System***

| ***The UNIVARIATE Procedure*** | |
| --- | --- |
| ***Variable: RS32*** |  |

| **Tests for Location: Mu0=0** | | | | |
| --- | --- | --- | --- | --- |
| **Test** | **Statistic** | | **p Value** | |
| **Student's t** | **t** | -0.72585 | **Pr > \|t\|** | 0.4724 |
| **Sign** | **M** | -0.5 | **Pr >= \|M\|** | 1.0000 |
| **Signed Rank** | **S** | -34 | **Pr >= \|S\|** | 0.5093 |

| **Analyse 2: FU1 VERSUS FU2** |
| --- |

***The SAS System***

| ***The MEANS Procedure*** |
| --- |

| **Variable** | **N** | **N Miss** | **Minimum** | **Lower Quartile** | **Median** | **Upper Quartile** | **Maximum** |
| --- | --- | --- | --- | --- | --- | --- | --- |
| RRSYST3 RRSYST4 | 28 28 | 0 0 | 100.00 98.00 | 120.00 120.00 | 130.00 134.00 | 140.00 143.50 | 165.00 160.00 |

***The SAS System***

| ***The UNIVARIATE Procedure*** | |
| --- | --- |
| ***Variable: RS43*** |  |

| **Tests for Location: Mu0=0** | | | | |
| --- | --- | --- | --- | --- |
| **Test** | **Statistic** | | **p Value** | |
| **Student's t** | **t** | 0.675353 | **Pr > \|t\|** | 0.5052 |
| **Sign** | **M** | 2 | **Pr >= \|M\|** | 0.5716 |
| **Signed Rank** | **S** | 25.5 | **Pr >= \|S\|** | 0.5706 |

| **Analyse 2: FU2 VERSUS FU3** |
| --- |

***The SAS System***

| ***The MEANS Procedure*** |
| --- |

| **Variable** | **N** | **N Miss** | **Minimum** | **Lower Quartile** | **Median** | **Upper Quartile** | **Maximum** |
| --- | --- | --- | --- | --- | --- | --- | --- |
| RRSYST4 RRSYST5 | 14 14 | 0 0 | 98.00 110.00 | 120.00 124.00 | 132.00 133.00 | 139.00 139.00 | 154.00 170.00 |

***The SAS System***

| ***The UNIVARIATE Procedure*** | |
| --- | --- |
| ***Variable: RS54*** |  |

| **Tests for Location: Mu0=0** | | | | |
| --- | --- | --- | --- | --- |
| **Test** | **Statistic** | | **p Value** | |
| **Student's t** | **t** | 1.55266 | **Pr > \|t\|** | 0.1445 |
| **Sign** | **M** | 1.5 | **Pr >= \|M\|** | 0.5811 |
| **Signed Rank** | **S** | 22.5 | **Pr >= \|S\|** | 0.1196 |

| **Analyse 2: FU3 VERSUS FU4** |
| --- |

***The SAS System***

| ***The MEANS Procedure*** |
| --- |

| **Variable** | **N** | **N Miss** | **Minimum** | **Lower Quartile** | **Median** | **Upper Quartile** | **Maximum** |
| --- | --- | --- | --- | --- | --- | --- | --- |
| RRSYST5 RRSYST6 | 14 14 | 0 0 | 110.00 100.00 | 122.00 107.00 | 133.00 128.50 | 139.00 134.00 | 170.00 160.00 |

***The SAS System***

| ***The UNIVARIATE Procedure*** | |
| --- | --- |
| ***Variable: RS65*** |  |

| **Tests for Location: Mu0=0** | | | | |
| --- | --- | --- | --- | --- |
| **Test** | **Statistic** | | **p Value** | |
| **Student's t** | **t** | -1.90867 | **Pr > \|t\|** | 0.0786 |
| **Sign** | **M** | -3 | **Pr >= \|M\|** | 0.1796 |
| **Signed Rank** | **S** | -31 | **Pr >= \|S\|** | 0.0511 |

| **Analysis diastolic bloodpressure** |  |
| --- | --- |
|  |  |
| **Analyse 1: Analysis all Patients** | |

***The SAS System***

| ***The MEANS Procedure*** |
| --- |

| **Variable** | **N** | **N Miss** | **Minimum** | **Lower Quartile** | **Median** | **Upper Quartile** | **Maximum** |
| --- | --- | --- | --- | --- | --- | --- | --- |
| RRDIAST1 RRDIAST2 RRDIAST3 RRDIAST4 RRDIAST5 RRDIAST6 | 61 52 44 29 15 17 | 0 9 17 32 46 44 | 60.00 50.00 60.00 66.00 71.00 70.00 | 70.00 70.00 70.00 78.00 80.00 78.00 | 80.00 80.00 80.00 84.00 83.00 85.00 | 85.00 80.00 85.00 89.00 92.00 90.00 | 108.00 100.00 100.00 100.00 104.00 97.00 |

| **Analyse 1: Analysis Patients at FU 4** |
| --- |

***The SAS System***

| ***The MEANS Procedure*** |
| --- |

| **Variable** | **N** | **N Miss** | **Minimum** | **Lower Quartile** | **Median** | **Upper Quartile** | **Maximum** |
| --- | --- | --- | --- | --- | --- | --- | --- |
| RRDIAST1 RRDIAST2 RRDIAST3 RRDIAST4 RRDIAST5 RRDIAST6 | 17 14 17 16 14 17 | 0 3 0 1 3 0 | 60.00 60.00 60.00 73.00 71.00 70.00 | 70.00 70.00 70.00 77.50 80.00 78.00 | 80.00 80.00 80.00 84.00 84.00 85.00 | 80.00 90.00 85.00 89.00 92.00 90.00 | 97.00 90.00 100.00 97.00 104.00 97.00 |

| **Analyse 1: Analysis Patients at FU 3** |
| --- |

***The SAS System***

| ***The MEANS Procedure*** |
| --- |

| **Variable** | **N** | **N Miss** | **Minimum** | **Lower Quartile** | **Median** | **Upper Quartile** | **Maximum** |
| --- | --- | --- | --- | --- | --- | --- | --- |
| RRDIAST1 RRDIAST2 RRDIAST3 RRDIAST4 RRDIAST5 RRDIAST6 | 15 12 15 14 15 14 | 0 3 0 1 0 1 | 60.00 60.00 60.00 73.00 71.00 70.00 | 70.00 70.00 70.00 77.00 80.00 75.00 | 80.00 80.00 80.00 84.00 83.00 85.50 | 87.00 80.00 80.00 89.00 92.00 90.00 | 97.00 90.00 100.00 97.00 104.00 97.00 |

| **Analyse 1: Analysis Patients at FU 2** |
| --- |

***The SAS System***

| ***The MEANS Procedure*** |
| --- |

| **Variable** | **N** | **N Miss** | **Minimum** | **Lower Quartile** | **Median** | **Upper Quartile** | **Maximum** |
| --- | --- | --- | --- | --- | --- | --- | --- |
| RRDIAST1 RRDIAST2 RRDIAST3 RRDIAST4 RRDIAST5 RRDIAST6 | 29 24 28 29 14 16 | 0 5 1 0 15 13 | 60.00 60.00 60.00 66.00 71.00 70.00 | 70.00 70.00 70.00 78.00 80.00 76.50 | 80.00 80.00 80.00 84.00 82.50 82.50 | 83.00 85.00 85.00 89.00 92.00 90.00 | 100.00 90.00 100.00 100.00 104.00 97.00 |

| **Analyse 1: Analysis Patients at FU 1** |
| --- |

***The SAS System***

| ***The MEANS Procedure*** |
| --- |

| **Variable** | **N** | **N Miss** | **Minimum** | **Lower Quartile** | **Median** | **Upper Quartile** | **Maximum** |
| --- | --- | --- | --- | --- | --- | --- | --- |
| RRDIAST1 RRDIAST2 RRDIAST3 RRDIAST4 RRDIAST5 RRDIAST6 | 44 39 44 28 15 17 | 0 5 0 16 29 27 | 60.00 50.00 60.00 73.00 71.00 70.00 | 70.00 70.00 70.00 79.00 80.00 78.00 | 80.00 80.00 80.00 84.50 83.00 85.00 | 88.00 80.00 85.00 89.50 92.00 90.00 | 100.00 100.00 100.00 100.00 104.00 97.00 |

| **Analyse 2: HOSPITAL ADMISSION VERSUS DISCHARGE** |
| --- |

***The SAS System***

| ***The MEANS Procedure*** |
| --- |

| **Variable** | **N** | **N Miss** | **Minimum** | **Lower Quartile** | **Median** | **Upper Quartile** | **Maximum** |
| --- | --- | --- | --- | --- | --- | --- | --- |
| RRDIAST1 RRDIAST2 | 52 52 | 0 0 | 60.00 50.00 | 70.00 70.00 | 80.00 80.00 | 87.00 80.00 | 108.00 100.00 |

***The SAS System***

| ***The UNIVARIATE Procedure*** | |
| --- | --- |
| ***Variable: RD21*** |  |

| **Tests for Location: Mu0=0** | | | | |
| --- | --- | --- | --- | --- |
| **Test** | **Statistic** | | **p Value** | |
| **Student's t** | **t** | -0.7336 | **Pr > \|t\|** | 0.4665 |
| **Sign** | **M** | -2.5 | **Pr >= \|M\|** | 0.5327 |
| **Signed Rank** | **S** | -61.5 | **Pr >= \|S\|** | 0.4298 |

| **Analyse 2: HOSPITAL DISCHARGE VERSUS FU1** |
| --- |

***The SAS System***

| ***The MEANS Procedure*** |
| --- |

| **Variable** | **N** | **N Miss** | **Minimum** | **Lower Quartile** | **Median** | **Upper Quartile** | **Maximum** |
| --- | --- | --- | --- | --- | --- | --- | --- |
| RRDIAST2 RRDIAST3 | 39 39 | 0 0 | 50.00 60.00 | 70.00 70.00 | 80.00 80.00 | 80.00 85.00 | 100.00 100.00 |

***The SAS System***

| ***The UNIVARIATE Procedure*** | |
| --- | --- |
| ***Variable: RD32*** |  |

| **Tests for Location: Mu0=0** | | | | |
| --- | --- | --- | --- | --- |
| **Test** | **Statistic** | | **p Value** | |
| **Student's t** | **t** | 0.34029 | **Pr > \|t\|** | 0.7355 |
| **Sign** | **M** | 1.5 | **Pr >= \|M\|** | 0.7011 |
| **Signed Rank** | **S** | 23 | **Pr >= \|S\|** | 0.5850 |

| **Analyse 2: FU1 VERSUS FU2** |
| --- |

***The SAS System***

| ***The MEANS Procedure*** |
| --- |

| **Variable** | **N** | **N Miss** | **Minimum** | **Lower Quartile** | **Median** | **Upper Quartile** | **Maximum** |
| --- | --- | --- | --- | --- | --- | --- | --- |
| RRDIAST3 RRDIAST4 | 28 28 | 0 0 | 60.00 73.00 | 70.00 79.00 | 80.00 84.50 | 85.00 89.50 | 100.00 100.00 |

***The SAS System***

| ***The UNIVARIATE Procedure*** | |
| --- | --- |
| ***Variable: RD43*** |  |

| **Tests for Location: Mu0=0** | | | | |
| --- | --- | --- | --- | --- |
| **Test** | **Statistic** | | **p Value** | |
| **Student's t** | **t** | 2.506144 | **Pr > \|t\|** | 0.0185 |
| **Sign** | **M** | 5 | **Pr >= \|M\|** | 0.0755 |
| **Signed Rank** | **S** | 90.5 | **Pr >= \|S\|** | 0.0181 |

| **Analyse 2: FU2 VERSUS FU3** |
| --- |

***The SAS System***

| ***The MEANS Procedure*** |
| --- |

| **Variable** | **N** | **N Miss** | **Minimum** | **Lower Quartile** | **Median** | **Upper Quartile** | **Maximum** |
| --- | --- | --- | --- | --- | --- | --- | --- |
| RRDIAST4 RRDIAST5 | 14 14 | 0 0 | 73.00 71.00 | 77.00 80.00 | 84.00 82.50 | 89.00 92.00 | 97.00 104.00 |

***The SAS System***

| ***The UNIVARIATE Procedure*** | |
| --- | --- |
| ***Variable: RD54*** |  |

| **Tests for Location: Mu0=0** | | | | |
| --- | --- | --- | --- | --- |
| **Test** | **Statistic** | | **p Value** | |
| **Student's t** | **t** | 0.917928 | **Pr > \|t\|** | 0.3754 |
| **Sign** | **M** | 1.5 | **Pr >= \|M\|** | 0.5488 |
| **Signed Rank** | **S** | 11 | **Pr >= \|S\|** | 0.3506 |

| **Analyse 2: FU3 VERSUS FU4** |
| --- |

***The SAS System***

| ***The MEANS Procedure*** |
| --- |

| **Variable** | **N** | **N Miss** | **Minimum** | **Lower Quartile** | **Median** | **Upper Quartile** | **Maximum** |
| --- | --- | --- | --- | --- | --- | --- | --- |
| RRDIAST5 RRDIAST6 | 14 14 | 0 0 | 71.00 70.00 | 80.00 75.00 | 84.00 85.50 | 92.00 90.00 | 104.00 97.00 |

***The SAS System***

| ***The UNIVARIATE Procedure*** | |
| --- | --- |
| ***Variable: RD65*** |  |

| **Tests for Location: Mu0=0** | | | | |
| --- | --- | --- | --- | --- |
| **Test** | **Statistic** | | **p Value** | |
| **Student's t** | **t** | -1.04389 | **Pr > \|t\|** | 0.3156 |
| **Sign** | **M** | -1 | **Pr >= \|M\|** | 0.7905 |
| **Signed Rank** | **S** | -14.5 | **Pr >= \|S\|** | 0.3821 |
