# Supplementary figures and images for: Outcome and clinical course of EHEC O104 infection in hospitalized patients: A prospective single center study
Source: PLoS One. 2018 Feb 8;13(2):e0191544. doi: 10.1371/journal.pone.0191544 (PMC5805174; doi:10.1371/journal.pone.0191544)

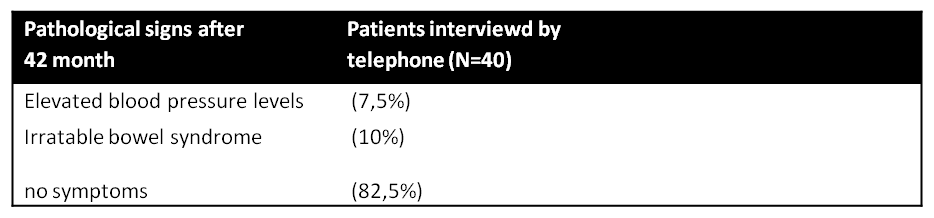

Supplement: S4 Fig — (TIF) [file pone.0191544.s004.tif]
